# Supplementary material for: One-Pot Decoration of Cupric Oxide on Activated Carbon Fibers Mediated by Polydopamine for Bacterial Growth Inhibition
Source: Materials (Basel). 2020 Mar 5;13(5):1158. doi: 10.3390/ma13051158 (PMC7085197; doi:10.3390/ma13051158)
Supplement: Supplementary file 1 [file materials-13-01158-s001.pdf]

materials

Supplementary Information

One-pot Decoration of Cupric Oxide on Activated Carbon Fibers Mediated by Polydopamine for the Inhibition of Bacteria Growth

Hangil Moon [1], Young-Chul Lee [2,*] and Jaehyun Hur [1,*]

1 Department of Chemical and Biological Engineering, Gachon University, Seongnam-si, Gyeonggi-do 13120, Korea; etenelsnow@naver.com
2 Department of BioNano Technology, Gachon University, Seongnam-si, Gyeonggi-do 13120, Korea
* Correspondence: dreamdbs@gachon.ac.kr (Y.-C.L.); jhhur@gachon.ac.kr (J.H.); Tel: +82-31-750-8751 (Y.-C.L.); +82-10-750-5593 (J.H.)

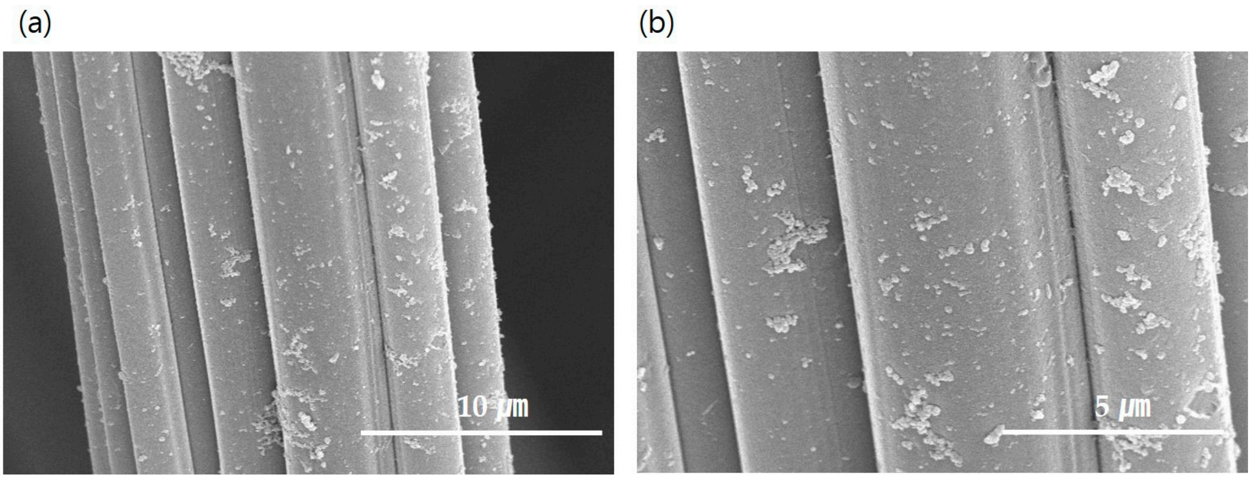

Figure S1. SEM images of ACF@PD at (**a**) low and (**b**) high magnification.

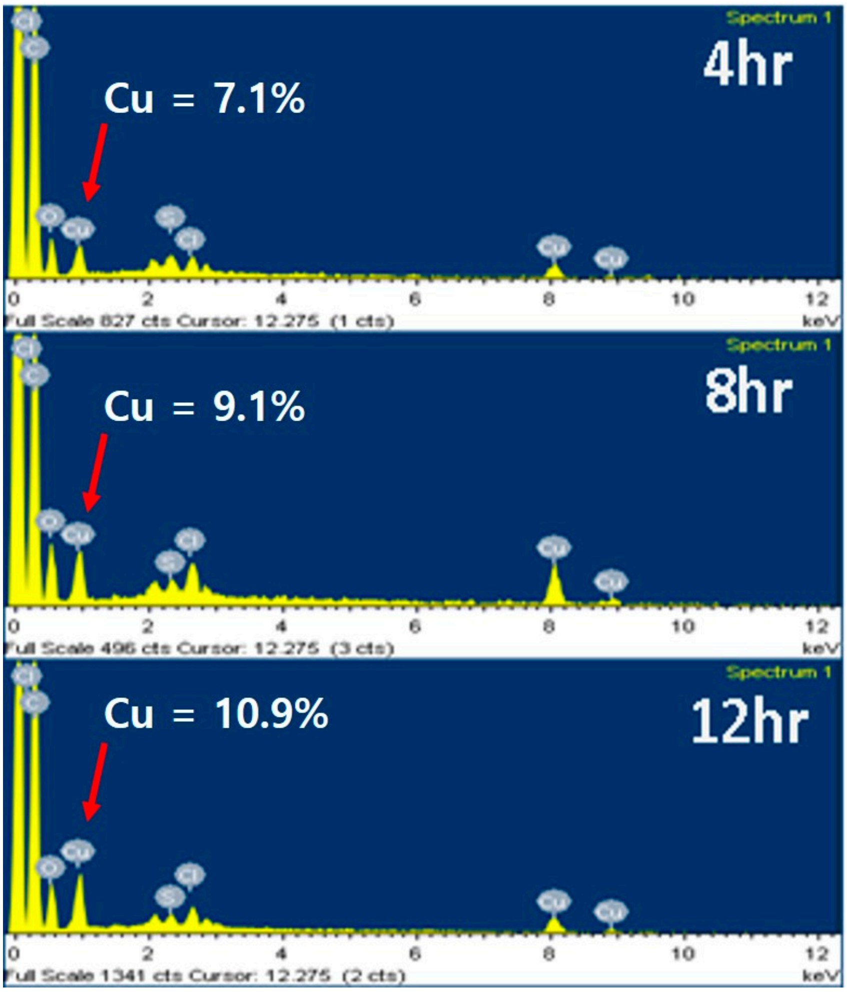

Figure S2. Cu content of ACF@PD-CuO with different coating time obtained from EDS analysis.

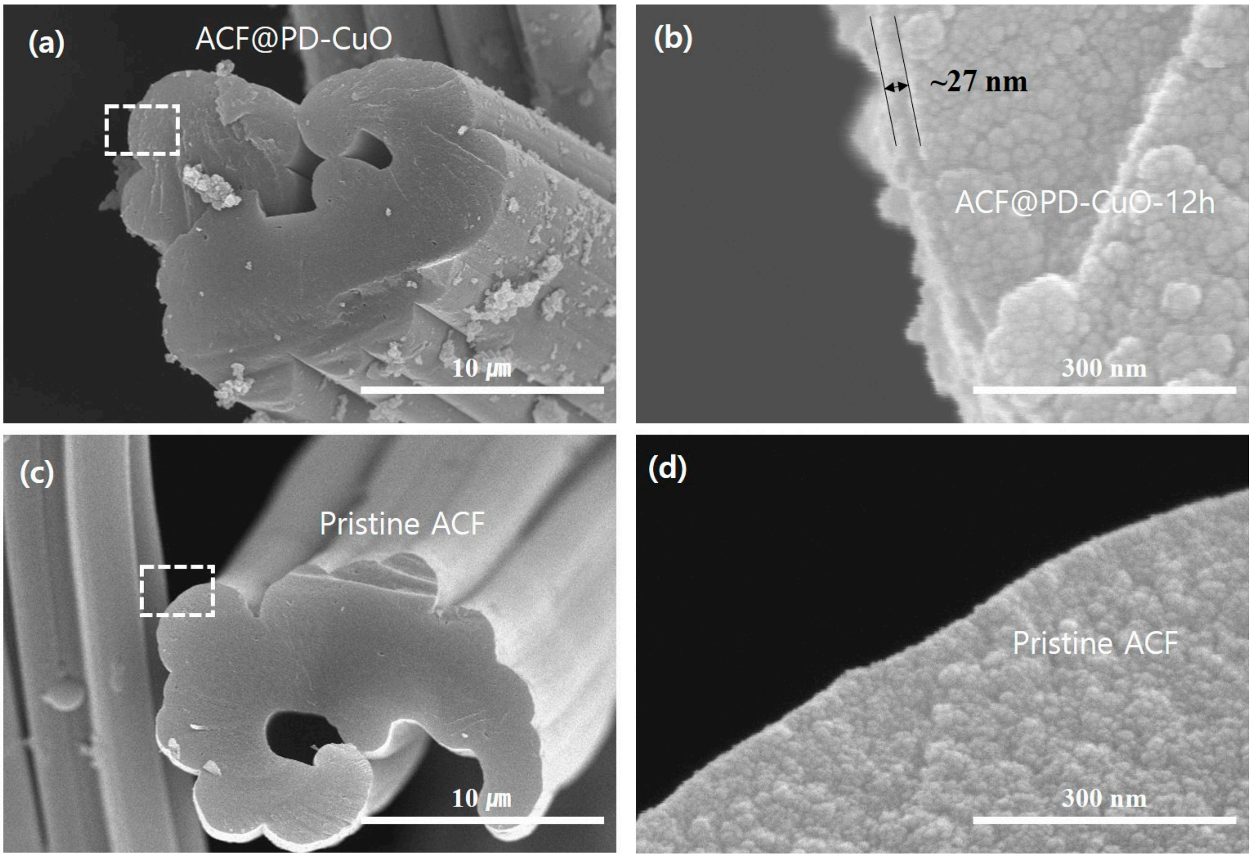

Figure S3. SEM images of ACF@PD-CuO-12h with (**a**) low- and (**b**) high-magnification and pristine ACF with (**c**) low- and (**d**) high-magnification to measure the precise coating thickness of PD-CuO.

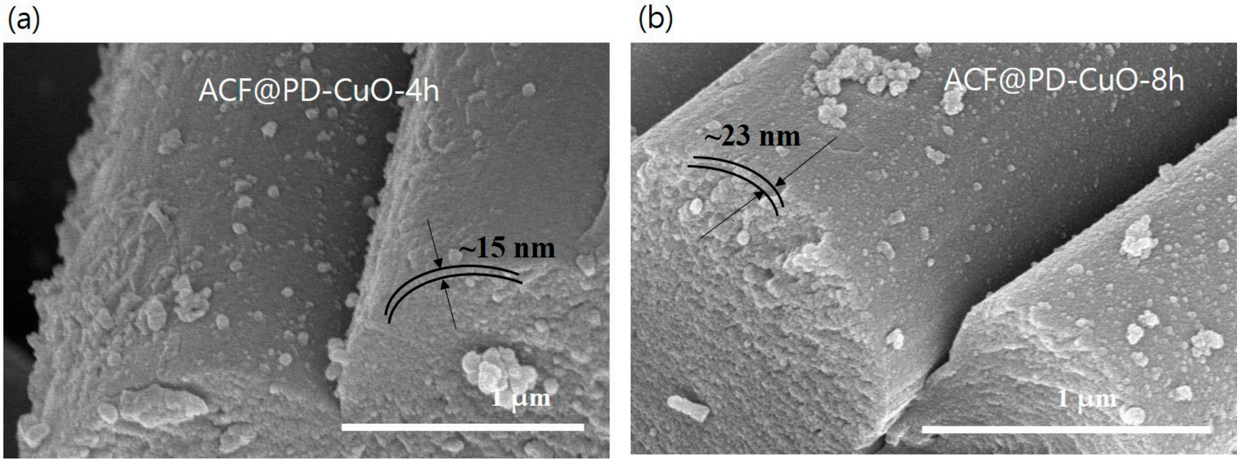

Figure S4. SEM images of (**a**) ACF@PD-CuO-4h and (**b**) ACF@PD-CuO-8h for measuring the coating thickness of PD-CuO.

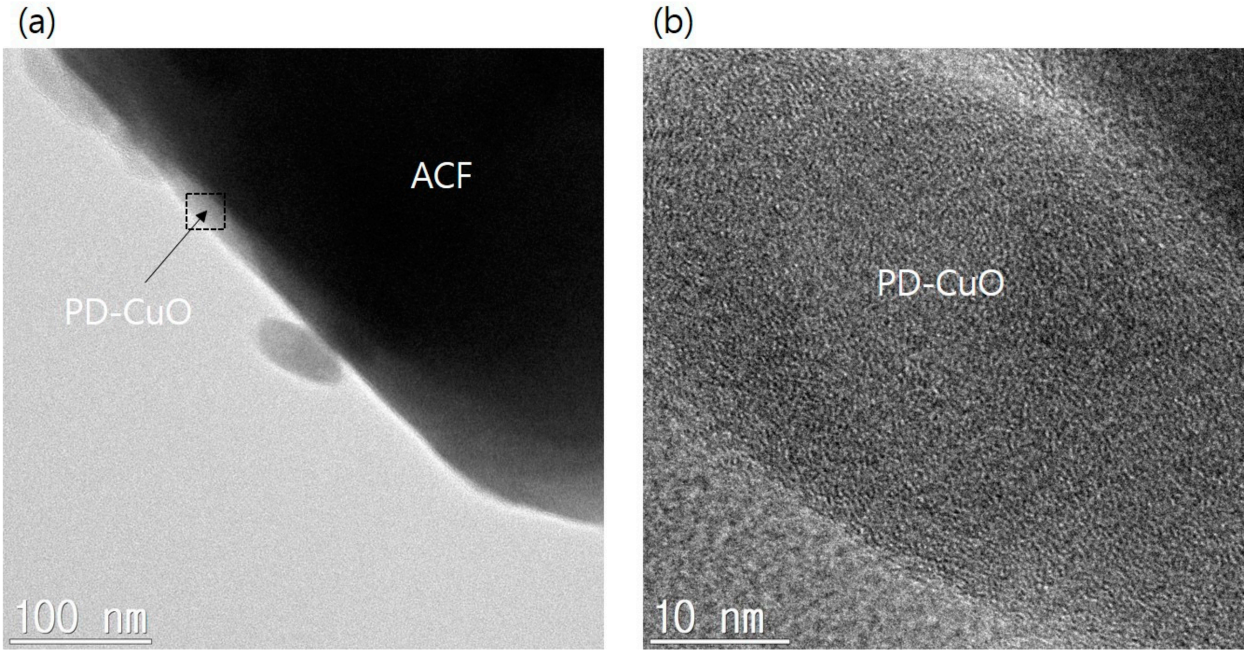

Figure S5. TEM images of ACF@PD-CuO-12h at (**a**) low and (**b**) high magnification.

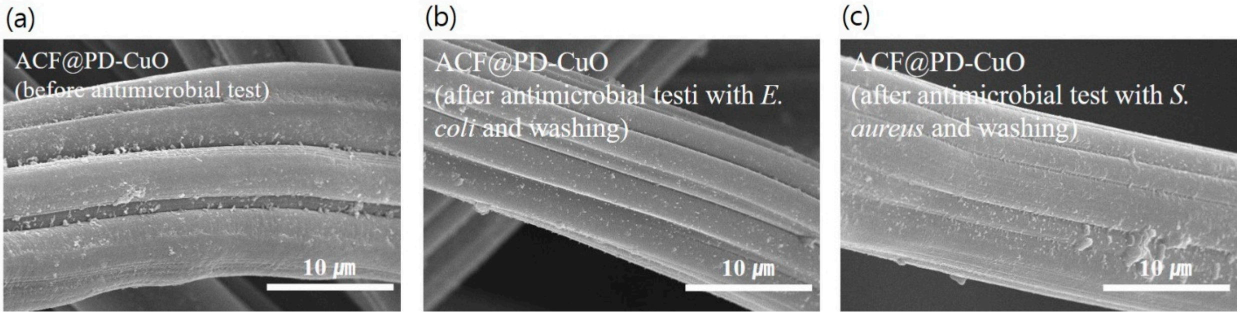

Figure S6. *Ex-situ* SEM images for ACF@PD-CuO (**a**) before the and after the antimicrobial test and washing: (**b**) *E-coli* and (**c**) *S-aureus*.
